# Allergic Contact Dermatitis From Dental Bonding Materials Containing Methacrylate: A Report of Two Cases

**DOI:** 10.7759/cureus.84395

**Published:** 2025-05-19

**Authors:** Abdulaziz Aljohani, Asma Aloufi, Ahmad A Qazali, Hamzah Babkair, Osama Qadiri, Hani T Fadel

**Affiliations:** 1 College of Dentistry, Taibah University, Medina, SAU; 2 Department of Substitutive Dental Sciences, College of Dentistry, Taibah University, Medina, SAU; 3 Department of Oral and Maxillofacial Diagnostic Sciences, College of Dentistry, Taibah University, Medina, SAU; 4 Department of Dentistry, Prince Mohammed Bin Abdulaziz Hospital, Medina, SAU; 5 Department of Preventive Dental Sciences, College of Dentistry, Taibah University, Medina, SAU

**Keywords:** allergic contact dermatitis (acd), dental adhesives, dental bonding, methacrylate, type iv hypersensitivity

## Abstract

Dental practitioners face occupational hazards, including allergic contact dermatitis (ACD) from materials like methacrylates found in dental bonding agents. ACD is a localized immune reaction causing dry skin, eczema, and sores. Despite using gloves, these chemicals can penetrate and cause skin reactions. The presented cases are of two dental practitioners who complained of signs and symptoms of ACD on the skin of their hands following regular restorative procedures. Later on, these complaints were diagnosed as ACD. The two male dentists developed ACD on the dorsal side of their hands due to improper handling of bonding materials. The allergens had penetrated despite using gloves, leading to localized rashes and itching. Diagnosed as a delayed type IV hypersensitivity reaction, both cases resolved with corticosteroids and improved handling. The exposure to methacrylate-containing dental bonding agents during routine procedures has the potential to develop ACD through direct or indirect skin contact. Increasing the awareness of the potential risks of methacrylate on the skin would consequently improve material handling techniques and identify appropriate methods for intervention and treatment in case the substance causes any adverse effects.

## Introduction

Dental practitioners are exposed to different occupational hazards, such as infection, radiation, percutaneous needle-stick accidents, and exposure to a variety of dental materials that may cause allergic contact dermatitis (ACD) [1]. This particular dermatitis is defined as a type of localized immune reaction that generally appears in the area of the skin that is in contact with the allergen. It can cause symptoms such as dry skin, patchy eczema, and chronic sores [2].

In dentistry, several dental materials could cause ACD, such as antimicrobials, rubber additives, preservatives, and methacrylates. Dental workers deal with a wide range of methacrylates present in different dental bonding agents, cements, direct dental restorations, and removable denture materials. It is estimated that methacrylate exposure accounts for 5% to 10% of all ACD reported incidents among dental professionals [2].

Dentists and their assistants often use the dorsal side of their non-dominant hand to hold different materials before introducing them to the working area. In addition, they use the same site to place the unfavorable excess materials. Endodontic lubricants, petroleum gel, impression materials, composite fillings, and dental bonding agents are examples of such materials. Even with following standard infection control measures, many studies showed that methacrylates can penetrate the gloves and reach the skin [3,4]. This is not surprising due to the misconception regarding the shielding effect of gloves. In comparison to latex and vinyl gloves, nitrile gloves have a longer breakthrough time, while thinner gloves and the presence of organic solvents reduce the required time till the allergen reaches the skin [3-5]. Additionally, there is a difference in breakthrough time within the acrylate types. While hydroxyethyl methacrylate (HEMA) needs only 6.3 minutes to penetrate the nitrile gloves, bisphenol A-glycidyl methacrylate (Bis-GMA) requires more than 180 minutes to reach the skin [5].

Based on our research, there is still a significant lack of awareness about the proper handling of bonding agents in the literature. More studies are needed to emphasize caution when working with bonding agents. The aim of the current report was to present two cases of dental practitioners who complained of signs and symptoms on the skin of their hands, later diagnosed as ACD, following regular dental restorative procedures.

## Case presentation

This report presents cases of two freshly graduated general dentists working at different private dental clinics in Al-Madinah Al-Munawwarah, Saudi Arabia. Both dentists were performing different surgical, periodontal, endodontic, and restorative procedures for less than a year. They were following the standard means of infection control, including hand disinfection and wearing gloves, masks, and gowns.

Approval to report these cases was sought from the Taibah University College of Dentistry, Research Ethics Committee (TUCD-REC) (Approval # TUCDREC/111024/HFadel; Date: 05/11/2024). Informed consent was obtained from the two participating dentists, and reassurance was given regarding the privacy of their personal data.

Case 1

**Figure 1:**
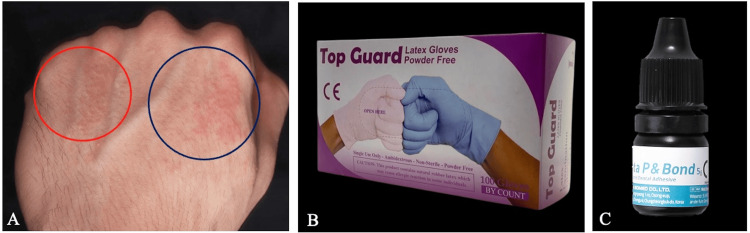
(A) Development of an erythematous patch, well-demarcated, with multiple papules on the dorsal side of the left hand (blue circle). A new lesion developed after changing the application site of the bonding agent (red circle). (B) Powder-free latex gloves (Top Guard). (C) Bonding agent (Meta P&Bond, Meta Biomed).

A 26-year-old male dentist noticed a skin lesion on the dorsal first web space of his left hand three weeks ago. The lesion started as dry skin with redness and itching, then the symptoms developed to eczematous rash, appearance of elevated papules, and an increase in the severity of itching (Figure 1A). The dentist had no significant medical history, and he was not on any medication, nor did he apply any hand cream or moisturizing agents. The dentist additionally mentioned that he performed many composite restorations during the previous month. While placing the restoration, he was applying uncured bonding agents on the gloves above the lesion area to lubricate the plastic instrument for easier composite manipulation. He was using powder-free latex gloves (Eiic, Top Guard, Kuala Lumpur, Malaysia) (Figure 1B) and a 5th-generation bonding system (Meta P&Bond, Meta Biomed, Cheongju-si, South Korea) (Figure 1C). According to the manufacturer, the bonding agent contained Bis-GMA, pyromellitic dianhydride glycerol dimethacrylate (PMGDM), 2-hydroxyethylmethacrylate (2-HEMA), and ethyl alcohol. To investigate the cause of the lesion, the dentist was directed to change his behavior by applying the uncured bonding agent on a glove above the third web space of the dorsal side of his non-dominant hand. Two weeks later, a skin rash started appearing on the new site (Figure 1A).

Case 2

**Figure 2:**
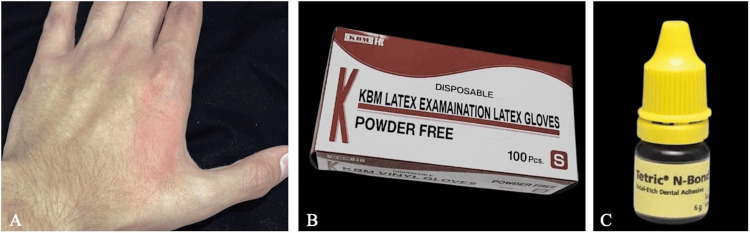
(A) Development of an erythematous patch, well-demarcated on the dorsal side of the left hand. (B) Powder-free latex gloves (KBM Co., Osaka, Japan). (C) Bonding agent (Tetric N-Bond, Ivoclar Vivadent).

A 27-year-old male dentist with no medical history or known allergies reported to his supervisor having an itchy rash localized to the dorsal first web space of his non-dominant hand (Figure 2A). During history-taking of the lesion, the patient mentioned that the lesion repeatedly appeared and subsided within two to three weeks, but the itching was milder in the previous episodes. Furthermore, the dentist confirmed that he applies a bonding agent over the gloves on the lesion area. He also mentioned that he used a micro-brush to remove the excess bonding agent and applied the excess over the glove. The dentist was using latex gloves (Figure 2B) and a 5th-generation bonding system as an adhesive (Tetric N-Bond, Ivoclar Vivadent, Zurich, Switzerland) (Figure 2C). The bonding agent contained phosphoric acid acrylate, HEMA, Bis-GMA, and urethane dimethacrylate (UDMA).

Diagnosis and treatment

**Figure 3:**
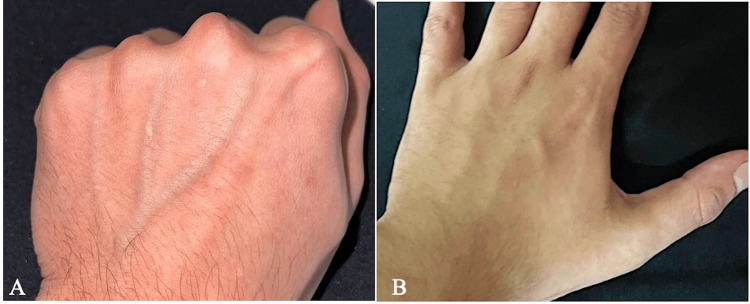
(A) The hand of the patient in the first case after three weeks of treatment. (B) The hand of the patient in the second case after three weeks of treatment.

After further investigation, the dermatologist diagnosed the two cases with ACD, a delayed type IV hypersensitivity reaction. In addition to instructing the two dentists, i.e., patients, to discontinue the habit, the dermatologist prescribed an over-the-counter topical corticosteroid, i.e., hydrocortisone 1% cream, to apply as a thin layer on the surface of the lesion, two to three times daily for 10 days. Within three weeks, the signs and symptoms subsided gradually in both cases, with no report of recurrence (Figure 3).

## Discussion

Applying dental bonding materials on the skin surface could cause ACD, even if the skin is protected by latex, vinyl, or nitrile gloves [4]. However, the type of gloves and the size of the methacrylate particles contribute to the duration of penetration [4,5]. Munksgaard [4] specifically mentioned that higher concentrations of methacrylate monomers increased glove permeability. However, regardless of the fact that the bonding agents’ safety data sheets contain a general warning on the use of safety measures, it did not specify the possibility of ACD symptoms [6].

When compared with other case reports, there are many similarities [5-7]. For example, difficulty in detecting the cause of the lesion was common in the discussed cases. This is because the primary diagnosis depends on the practitioner's history, and it can be mixed with different types of allergens that could cause the same symptoms. One of the significant features that may help reach a correct diagnosis is the location of the lesion. In the majority of the reported cases, the location of the lesion was the same [5-7]. This is owed to the common behavior among dental workers to use the dorsal side of the non-dominant hand to facilitate the application or removal of dental materials. While it is a safe technique with some materials, it is potentially dangerous to smear dental resin adhesives over the gloves or skin, either in a direct or indirect way. Moreover, when adhesives are contaminated or mixed with the gloves' powder, they may decrease their mechanical and/or physical properties [8].

Interestingly, erythematous plaques with itching and burning sensations were the common signs and symptoms among the current cases [5-7]. Such symptoms appeared with the majority of reported ACD cases, corresponding to a delayed type IV hypersensitivity reaction. However, the signs and symptoms in one case were more aggressive and were displayed as localized tense blisters. This may have been in part due to misdiagnosis and consequent delayed intervention [7]. After trying a number of treatment strategies, such as applying moisturizers on the affected areas, the signs in the reported cases were resolved within one to three weeks. The first line of treatment in all cases was to quit the habit, and corticosteroid cream was used in some cases to accelerate the healing process [6]. This points to the importance of following a systematic investigative approach to reach an accurate diagnosis, facilitate rapid and efficient intervention, avoid initially preventable complications, and spare unnecessary added costs and manpower.

Lastly, the reported cases are for freshly graduated dentists who may not be fully aware of the dangers associated with handling acrylate-containing products and the limitations of glove protection against such allergens. Moreover, fresh graduates may spend more time in restoring teeth than experienced practitioners, consequently increasing the exposure time to the allergens in the process. However, even if the procedure time was short, some articles reported that methyl methacrylate (MMA) particles need only three minutes to penetrate the gloves and start harming the underlying skin [9]. Using a double-gloving technique to extend the protection from chemical allergens has been suggested [6,10].

## Conclusions

This report presented two cases of dental practitioners who suffered from signs and symptoms corresponding to ACD following daily restorative dental procedures.

These cases suggest that methacrylate-containing bonding agents have the potential to cause ACD when they come into direct or indirect contact with the skin. Accordingly, it is important to raise awareness among dental practitioners, assistants, technicians, and denture wearers to avoid such a problem.
